# GASTROINTESTINAL STROMAL TUMOR: OUTCOMES OF THE PAST DECADE IN A REFERENCE INSTITUTION IN SOUTHERN BRAZIL

**DOI:** 10.1590/0102-672020210002e1658

**Published:** 2022-06-17

**Authors:** Eduardo Morais EVERLING, Daniele MARCHET, Natália Marchet DE-ANTONI, Bruna Bley Mattar ISBERT, Gustavo Vasconcelos ALVES, Tomaz de Jesus Maria GREZZANA-FILHO

**Affiliations:** 1 Department of General Surgery, Hospital Nossa Senhora da Conceição (HNSC) - Porto Alegre - RS - Brazil;; 2 Digestive System Surgery, Hospital de Clínicas de Porto Alegre (HCPA), - Porto Alegre - RS - Brazil;; 3 Department of Clinical Cancerology, Hospital Nossa Senhora da Conceição (HNSC), - Porto Alegre - RS - Brazil;; 4 Department of Clinical Cancerology, Hospital de Clínicas de Porto Alegre (HCPA), - Porto Alegre - RS - Brazil.

**Keywords:** Gastrointestinal Stromal Tumors, Imatinib Mesylate, Surgical Oncology, Digestive System Surgical Procedures, Survival Analysis, Tumores do Estroma Gastrointestinal, Mesilato de Imatinib, Oncologia Cirúrgica, Procedimentos Cirúrgicos do Aparelho Digestivo, Análise de Sobrevida

## Abstract

Gastrointestinal stromal tumor (GIST) is the most common mesenchymal neoplasm of the digestive tract and has a wide variation in biological behavior; surgical resection remains the main form of treatment.

**AIM:**

This study aimed to analyze clinicopathological characteristics and survival of patients with GIST in a reference institution for oncological diseases.

**METHODS:**

An observational, longitudinal, and retrospective study of patients diagnosed with GIST from January 2011 to January 2020 was carried out by analyzing epidemiological and clinical variables, staging, surgical resection, recurrence, use of imatinib, and curves of overall survival (OS) and disease-free survival (DFS).

**RESULTS:**

A total of 38 patients were included. The majority (58%) of patients were males and the median age was 62 years. The primary organs that were affected by this tumor were stomach (63%) and small intestine (17%). Notably, 24% of patients had metastatic disease at diagnosis; 76% of patients received surgical treatment and 13% received neoadjuvant treatment; and 47% of patients received imatinib as adjuvant or palliative therapy. Tumor recurrence was 13%, being more common in the liver. The 5-year OS was 72.5% and DFS was 47.1%. The operated ones had better OS (87.1% *vs.* 18.5%) and DFS (57.1% *vs.* 14.3%) in 5 years. Tumor size ≥5 cm had no difference in OS at 5 years, but DFS was 24.6%, when compared with 92.3% of smaller tumors. Patients who were undergoing neoadjuvant therapy and/or using imatinib did not show any significant differences.

**CONCLUSIONS:**

Surgical treatment with adequate margins allows the best gain in survival, and the use of imatinib in more advanced cases has prognostic equity with less advanced-stage tumors. Treatment of metastatic tumors seems promising, requiring further studies.

## RESUMO - RACIONAL

O Tumor estromal gastrointestinal (*Gastrointestinal stromal tumor* - GIST) é a neoplasia mesenquimal mais comum do trato digestivo, possui comportamento biológico variado e a principal forma de tratamento é a ressecção cirúrgica.

**OBJETIVO:**

analisar as características clínico-patológicas e a sobrevida de pacientes com GIST em uma instituição de referência para doenças oncológicas.

**MÉTODOS:**

Foi realizado um estudo observacional, longitudinal e retrospectivo de pacientes com diagnóstico de GIST de janeiro de 2011 a janeiro de 2020, analisando variáveis epidemiológicas e clínicas, estadiamento, ressecção cirúrgica, recidiva, uso de imatinibe e curvas de sobrevida global (SG) e sobrevida livre de doença (SLD).

**RESULTADOS:**

foram incluídos 38 pacientes, a maioria (58%) do sexo masculino, idade mediana de 62 anos. Os principais órgãos primários foram estômago (63%) e intestino delgado (17%). 24% tinham doença metastática ao diagnóstico. 76% receberam tratamento cirúrgico e 13% tratamento neoadjuvante. 47% dos pacientes receberam Imatinib como terapia adjuvante ou paliativa. A recorrência tumoral foi de 13%, mais comum no fígado. SG de 5 anos foi de 72,5% e SLD 47,1%. Os operados tiveram melhor SG (87,1% *vs.* 18,5%) e SLD (57,1% *vs*. 14,3%) em 5 anos. O tamanho do tumor igual ou maior que 5 cm não teve diferença na SG em 5 anos, mas SLD foi de 24,6%, em comparação com 92,3% dos tumores menores. Pacientes em terapia neoadjuvante e/ou em uso de imatinibe não apresentaram diferenças significativas.

**CONCLUSÕES:**

O tratamento cirúrgico com margens adequadas permite o melhor ganho de sobrevida, e o uso de Imatinibe em casos mais avançados tem equidade prognóstica com tumores em estágio menos avançado. O tratamento de tumores metastáticos parece promissor, necessitando de mais estudos.

## INTRODUCTION

A gastrointestinal stromal tumor (GIST) is the most common mesenchymal neoplasia of the digestive tract. Such tumors originate in the Cajal cells of the lamina propria, which are present in the gastrointestinal tube and perform motility-related functions^16^ ^,^ ^19^. Since the recognition of mutations of the KIT and PDGFRA genes and clinical application of the use of anti-tyrosine kinase agents such as imatinib, there have been significant advances in the understanding of the clinical and molecular characteristics of this neoplasia. However, such tumors have a wide variation in biological behavior. Surgery remains the main form of treatment, even in the age of target therapies ^3^. In this study, we analyzed clinicopathological characteristics and survival of localized and metastatic tumors in a single public institution of reference on the treatment of oncological diseases.

## METHODS

An observational, longitudinal, and retrospective study was conducted. All the patients with a diagnosis of GIST obtained through histopathological analysis and confirmed by immunohistochemistry from January 2011 to January 2020 were included in the study. The data were obtained through the review of hospital records, with the analysis of epidemiological and clinical variables; clinical and pathological staging; surgical resection; recurrence indices; imatinib use; and the curves of overall survival (OS), defined as the absence of death in 5 years, and disease-free survival (DFS), defined as the absence of recurrence or death in 5 years. This study was approved by the Institutional Research Ethics Committee under number 2,080,502.

The statistical analysis was performed using the SPSS Statistics, version 22.0 software. The survival analysis was carried out using the Kaplan-Meier method to assess OS and DFS in the 5-year period and using the log-rank (Mantel-Cox) test to compare the variables. Risk and multivariate analyses were obtained through the Cox regression test.

The risk and prognosis assessment was performed through the Fletcher’s classification, which establishes two factors as prognostic parameters of patients with GISTs: one is macroscopic (tumor size) and the other is microscopic (mitotic index) ^8^. This combination resulted in a system that classifies tumors into different degrees of risk, with a tumor being considered high risk when its size is >5 cm with five mitoses in 50 high-power fields (HPF), its size is >10 cm with any mitotic index, or it has over 10 mitoses in 50 HPF regardless of the size.

## RESULTS

Thirty-eight patients with GISTs were diagnosed in the analysis period. The disease proved to be more frequent in male (58%) and white (92%) individuals. The median age at the time of diagnosis was 62 years, varying from 22 to 83 years. There was a previous diagnosis of neoplasia for 21% of the patients. As per the ECOG scale, 53% of the cases were classified as having a good functional capacity (active, without restrictions). The stomach (63%) was the most affected organ, followed by the small intestine (17%). The most common symptom reported during the analysis was abdominal pain, which was identified in 45% of the cases. For 24% of the individuals, the tumor lesion was detected with the help of a CT scan that was performed for another purpose. During the initial diagnosis, 24% of patients had metastatic disease. The median tumor size was 5.6 cm (0.2-22.4 cm). The demographic and clinicopathological characteristics are described in part I of Table 1.

**Table 1 -:** Demographic and clinicopathological characteristics and outcomes of patients diagnosed with GIST.

		N (%)
I - Demographic and clinicopathological characteristics of patients diagnosed with GIST		
Gender	Male Female	22 (58) 16 (42)
Race	White Brown Black	35 (92) 2 (5) 1 (3)
Previous cancer	Yes No	8 (21) 30 (79)
ECOG* Scale	0 1 2 3 4 No information	20 (53) 6 (16) 5 (13) 2 (5) 2 (5) 3 (8)
Primary GIST location	Stomach Duodenum Small bowel Liver Mesentery Rectum Adrenal Ovary	24 (63) 1 (3) 7 (17) 1 (3) 2 (5) 1 (3) 1 (3) 1 (3)
Clinical presentation	Abdominal pain Nausea/emesis Gastrointestinal bleeding Acute abdomen Abdominal mass Incidental finding	17 (45) 9 (24) 9 (24) 6 (16) 8 (21) 9 (24)
KIT/CD117	Positive Weak positive Negative	35 (92) 1 (3) 2 (5)
Mitotic index	≤5/50 HPF >5/50 HPF No information	25 (66) 7 (17) 6 (16)
Tumor size	<5 cm ≥5 cm No information	14 (37) 21 (55) 3 (8)
Staging	IA IB IIA IIB IIIA IIIB IV No information	8 (21) 2 (5) 2 (5) 2 (5) 3 (8) 5 (13) 13 (35) 3 (8)
Metastatic disease at diagnosis	Yes No No information	9 (24) 25 (66) 4 (10)
Metastatic site	Liver Peritoneum Mesentery	6 (66) 3 (33) 2 (22)
**II - Treatment and outcomes of patients diagnosed with GIST**		
Surgery	Yes No	29 (76) 9 (24)
Type of ressection	R0 R1 R2	25 (86) 1 (4) 3 (10)
Imatinib	Yes No	18 (47) 20 (53)
Neoadjuvant therapy	Yes No No information	4 (10) 32 (85) 2 (5)
Adjuvant therapy	Yes No No information	11 (29) 8 (21) 19 (50)
Paliative care	Yes No No information	5 (13) 13 (34) 20 (53)
Tumor rupture	Yes No No information	4 (10) 33 (87) 1 (3)
Tumor recurrence	Yes No No information	5 (13) 28 (74) 5 (13)
Recurrence site	Liver Peritoneum Mesentery Esophagus	3 (60) 1 (20) 1 (20) 1 (20)
Death	Yes No No information	10 (26) 24 (64) 4 (10)

ECOG: Eastern Cooperative Oncology Group; HPF: high-power fields; R0: absence of residual tumor (clear margins); R1: microscopic residual tumor (compromised margins); R2: macroscopic residual tumor

In total, 76% of the patients were submitted to surgical treatment with the resection being considered R0 for 86% of the cases. Neoadjuvant treatment was performed in four (13%) cases. Imatinib was prescribed to 18 (47%) patients, being used as adjuvant therapy in 11 (29%, median of 36 months of use) patients and as palliative therapy in 5 cases (13%, median of 29 months of use). In 13% of the cases, tumor recurrence was diagnosed after the treatment of the primary neoplasia, with a median of 48 months after surgery. In three cases, the most common site of recurrence was found to be the liver. Ten (26%) patients died during the follow-up period. The median follow-up time was 24 months, with a variation from 0 to 163 months. Details of such treatment variables and outcomes are presented in part II of Table 1.

**Figure 1 -:**
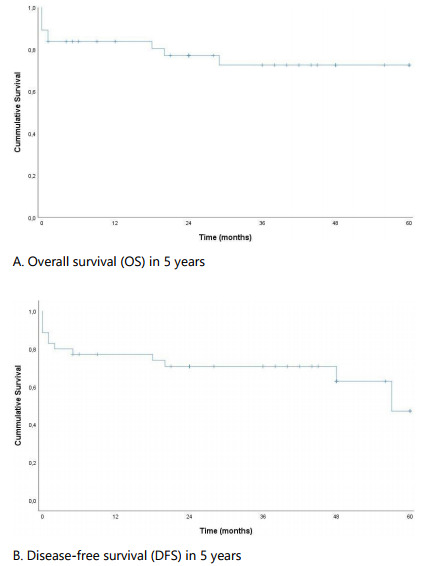
Overall survival (A) and disease-free survival (B) in 5 years of patients diagnosed with gastrointestinal stromal tumors.

An OS in 5 years of 72.5% was observed in the analysis sample, while the DFS was 47.1%. The OS and DFS curves are presented in Figure 1.

The number of patients in the OS analysis according to the time was as follows: 0 months - 37; 12 months - 26; 24 months - 22; 36 months - 16; 48 months - 9; 60 months - 5. The number of patients in the DFS analysis according to the time was as follows: 0 months - 35; 12 months - 24; 24 months - 21; 36 months - 16; 48 months - 9; 60 months - 3.

**Figure 2 -:**
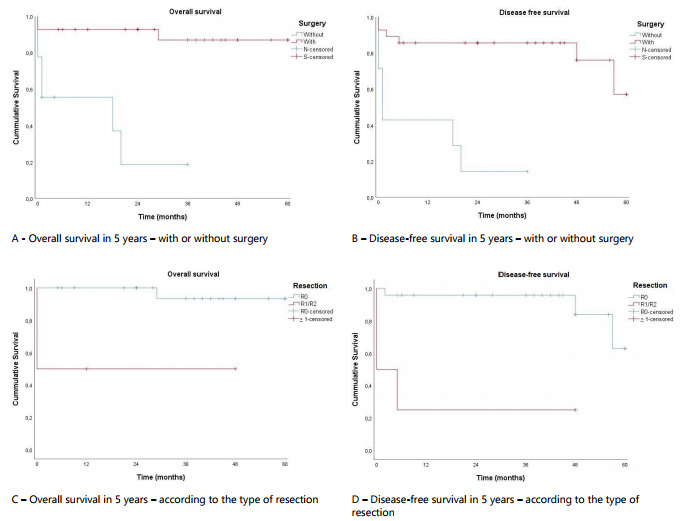
Kaplan-Meier charts related to surgical treatment for patients with GIST. (A) Overall survival in 5 years - with or without surgery; (B) disease-free survival in 5 years - with or without surgery; (C) Overall survival in 5 years - according to the type of resection; (D) Disease-free survival in 5 years - according to the type of resection.

When analyzing the variables with impact on the survival of the patients diagnosed with GIST, it was observed that patients submitted to surgical treatments presented a significant increase in OS and DFS in 5 years compared to patients treated without resection of the primary tumor (87.1% *vs.* 18.5%, p<0.001 and 57.1% *vs.* 14.3%, p<0.001, respectively) (Figures 2A and 2B). The OS and DFS in 5 years were significantly higher in patients submitted to R0 resection, compared to patients with micro or macroscopic residual disease (R1 and R2) (93.3% *vs.* 50%, p=0.002 and 62.9% *vs.* 25%, p<0.01, respectively) (Figures 2C and 2D). The survival curves related to surgical treatment are presented in Figures 2A-2D.

The difference in OS (Figure 2A) and DFS (Figure 2B) in 5 years between patients submitted to surgery or not proved to be significant. The OS (Figure 2C) and DFS (Figure 2D) in 5 years as per the type of resection in patients submitted to surgical treatment also proved to be significant.

**Figure 3 -:**
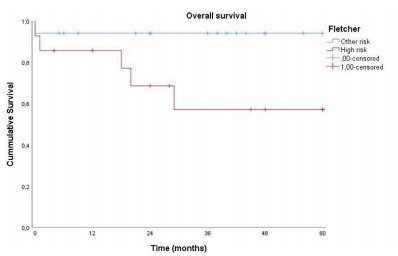
Kaplan-Meier chart relating overall survival in 5 years to the Fletcher’s classification.

Patients classified as high risk, according to the Fletcher’s classification, presented OS in 5 years, which was significantly lower than the other risk groups (intermediate, low, and very low) (p=0.046), as demonstrated in Figure 3.

The very low, low, and intermediate risks were compared with the high risk of malignancy. Patients classified as high risk presented OS in 5 years of 57.1%, while the other patients presented OS in the same period of 94.1%.

**Figure 4 -:**
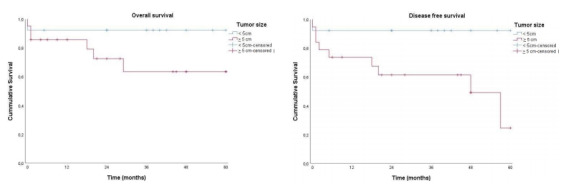
Kaplan-Meier charts relating overall and disease-free survival in 5 years to tumor size.

Individuals with tumor size ≥5 cm did not present differences in OS in 5 years compared to the patients with tumors <5 cm (p=0.130). However, the DFS was 24.6% for the patients with tumors >5 cm and 92.3% for the patients with tumors <5 cm. This difference was significant (p=0.04) and is demonstrated in Figure 4.

The OS in 5 years was 92.3% for patients with tumor size <5 cm and 63.5% for patients with tumor size ≥5 cm. This difference was not statistically significant (p=0.13). The DFS in 5 years was 92.3% for patients with tumor size <5 cm and 24.6% for patients with tumor size ≥5 cm. This difference was statistically significant (p=0.04).

No significant difference in OS or DFS was observed according to the tumor grade (defined from the mitotic index, considered high grade if there were over 5 mitoses per 50 HPF and low grade if lower than or equal to 5 mitoses per 50 HPF; p=0.715).

Patients submitted to neoadjuvant treatment and/or using imatinib did not present significant differences in OS and DFS (p=0.954 and p=0.182, respectively). However, 55.6% of the individuals who used imatinib were staged with grade IV, and 17.6% of the cases that did not use imatinib had the same staging. The assessment of this parameter showed a significant difference (p=0.02).

For individuals with metastatic disease upon diagnosis, no difference was observed in the 5-year survival compared to the non-metastatic disease (55.6% *vs.* 78.3%, p=0.06).

The multivariate analysis using the Cox regression test demonstrated an increase in the risk of adverse outcomes and mortality in 5 years of follow-up for patients who were not submitted to surgery (RR 12.99, 95%CI 2.36-71.38, p=0.003). The variables of tumor size and metastatic disease upon diagnoses were not significant.

## DISCUSSION

GISTs are rare but still represent the most common mesenchymal neoplasia of the digestive tract ^21^. Global data from the past decades have demonstrated a huge variability regarding the incidence of this neoplasia, with incidences being reported varying from 4 cases per million in North America to 22 cases per million in countries such as China, South Korea, and Norway ^21^. However, the improvement in the notification of such cases, the correction of the differentiation between malignant and benign cases, and the use of specific registration systems have improved the epidemiological understanding of this neoplasia. The most recent data suggest an incidence of eight cases per million per year, which is consistent with several European studies ^10^.

There are few reports of case series in Brazil, with outcomes from the past decade, which have not yet been described. Given the improvement in the clinical and surgical treatment of these neoplasias in the past decades, better knowledge of the current reality becomes indispensable. As expected, the stomach was the most affected organ in our series, followed by the small intestine. The average age was similar to those of global studies ^1^ ^,^ ^5^ ^,^ ^9^ ^,^ ^15^ ^,^ ^21^ ^.^ A significant number of cases diagnosed with tumor size >5 cm (55%) and grade IV staging (35%) were observed with factors considered of worse prognosis. This is consistent with other series published in Brazil, which reported an average size >10 cm ^7^ ^,^ ^12^. The association of GISTs with other neoplasias is common and was also observed in our casuistry, as well as in a relevant number of cases with distant metastases, similar to other studies ^17^.

Compared with other case series, we observed a higher rate of R0 resections in this series, which may explain OS and DFS rates that were more favorable than those found in studies performed in the decades that preceded the current series (Table 2).

**Table 2 -:** Comparison of a series of national cases of gastrointestinal stromal tumors.

	Location	N	Tumor size (mean) (cm)	Surgery (%)	Resection R0 (%)	OS in 5 years (%)	DFS in 5 years (%)
Present study	Porto Alegre, RS	38	5.6	76	86	72.5	47.1
Linhares et al. 2011^7^	Rio de Janeiro, RJ	146	11.8	93.8	70.8	59	50
Dos Santos Junior et al. 2012^12^	Fortaleza, CE	45	11.7	97.8	77.8	60	39

In various studies, tumor size and mitotic index are usually prognostic factors of this neoplasm ^5^. However, in this series, when such parameters were analyzed individually, they demonstrated no differences in the OS. However, according to Fletcher’s risk classification, the conjugated analysis demonstrated a different scenario. Our data demonstrated a significant difference between the tumors classified as very low, low, and intermediate grades, which reached an OS in 5 years of 94.1%, and the tumors considered high risk, which had a significantly lower OS in 5 years (57.1%). These findings were also observed by Linhares et al.^12^, who detected rates of OS in 5 years in these groups of 76% and 49%, respectively. In terms of DFS, the tumor size >5 cm was a significant parameter in this series, similar to other studies ^17^. Other authors used the Miettinen scale, which evaluates, besides the two factors already mentioned, the organ affected by the tumor ^14^.

After the introduction of imatinib in 2001 into the therapeutic arsenal as first line of treatment and, later, sunitinib and regorafenib in cases of resistance to the first line, there has been a consistent improvement in the treatment of this neoplasia. Besides its use as an adjuvant after surgical treatment, imatinib also has an important role as a neoadjuvant in situations of locally advanced yet potentially resectable diseases and as a palliative agent in cases that were considered unresectable ^4^ ^,^ ^18^. In the past decade, there was a significant advance in the understanding of the molecular alterations of this neoplasia, and it is currently own that the KIT gene is present in 80-90% of the cases, with mutations of exon 11 of the KIT gene being observed in two-thirds of the cases and of exon 9 in 8-10% of the cases, the latter being associated with tumors of the small intestine and colon ^4^. As other examples, the deletions involving codons 557 and 558 of this gene are particularly involved in worse prognosis compared to punctual mutations ^13^. In turn, the mutational variant derived from the Platelet-Derived Growth Factor Receptor Alpha (PDGFRA) occurs in approximately 10% of the cases and is generally observed in the stomach ^2^. Our data initially demonstrated that using imatinib was not related to a significant improvement in OS and DFS. However, a more detailed analysis demonstrated that the adjuvant therapy was mostly destined to the cases with more advanced staging (stage IV), with those who used imatinib having the same R0 resection indices and similar mitotic indices than those who did not. Hence, the effect of using imatinib allowed OS and DFS of the stage IV cases similar to those with less advanced stages, confirming the positive action of the drug. Similarly, imatinib was used as a neoadjuvant in cases with locally advanced diseases and less favorable staging. Even so, the outcomes of the individuals who underwent neoadjuvant treatment were similar to those of individuals who did not use this treatment, thus demonstrating the benefit of using imatinib in the selected cases. These findings agree with those of other authors who previously demonstrated the positive effect of imatinib as a neoadjuvant after the treatment of GISTs in advanced stages^6^.

Despite these advances, surgical treatment remains the only therapy with a possibility of a cure. Typically, GISTs rarely disseminate to regional lymph nodes, and formal lymphadenectomy is not usually indicated except in cases of enlarged lymph nodes adjacent to the involved organ. A surgical technique with the least possible manipulation (“no-touch”) is recommended to preserve the tumor capsule and avoid the peritoneal dissemination at all costs, given that the rupture is related to survival impairment. Resection with expanded margins is unnecessary and not related to better results but may increase the complications index. According to various authors, local resection with R0 margins is the most important factor regarding OS in localized GISTs ^20^. Our findings confirm a strong relationship between the cases submitted to surgery with R0 margins and a significant improvement in OS and DFS. In contrast, in this series, a resection with positive margins meant an average reduction of 42 months in OS. The strong effect of surgery, even with positive margins, was also confirmed in the multivariate analysis, being the only parameter significantly associated with survival in the current series.

Despite the restricted number of cases, an interesting finding was similar OS and DFS in the cases of metastatic GIST and the cases without metastasis. In our series, 44% of the metastatic tumors were submitted to R0 resection, and the metastatic tumor was not a prognostic factor in the univariate analysis. According to the current understanding, a metastatic disease restricted to one or two organs with possibilities of resection (e.g., liver, peritoneum) does not impede the surgical treatment and may confer OS similar to non-metastatic cases ^11^. However, due to the restricted number of cases assessed, caution is recommended in the interpretation of this result, which requires confirmation.

The limitations of this study are the retrospective nature and a limited number of study individuals. Considering these are rare tumors and also the absence of a specific registration of this neoplasia, the data gain relevance and demonstrate an advance in terms of survival in the past decade compared to other periods.

## CONCLUSIONS

The surgical treatment of the GISTs with appropriate margins allows the best gain in terms of survival, with the use of imatinib in the more advanced staging cases obtaining a benefit to the point of reaching prognostic equity with tumors in less advanced stages. The treatment of metastatic tumors seems promising, yet needs a directed assessment to confirm the findings of this series.
